# Patient Preferences for Direct-to-Consumer Telemedicine Services: Replication and Extension of a Nationwide Survey

**DOI:** 10.2196/51056

**Published:** 2024-11-27

**Authors:** Julia Ivanova, Hattie Wilczewski, Farina Klocksieben, Mollie Cummins, Hiral Soni, Triton Ong, Janelle Barrera, Jillian Harvey, Nathaniel O'Connell, James McElligott, Brandon Welch, Brian Bunnell

**Affiliations:** 1 Doxy.me Research, Doxy.me, Inc., 3445 Winton Pl, #114, Rochester, NY, 14623, United States, 1 6025618861; 2 Research Methodology and Biostatistics Core, Morsani College of Medicine, University of South Florida, Tampa, FL, United States; 3 Department of Biomedical Informatics, College of Nursing and Spencer Fox Eccles School of Medicine, University of Utah, Salt Lake City, UT, United States; 4 Department of Psychiatry and Behavioral Neurosciences, Morsani College of Medicine, University of South Florida, Tampa, FL, United States; 5 Healthcare Leadership and Management, College of Health Professions, Medical University of South Carolina, Charleston, SC, United States; 6 Department of Biostatistics and Data Science, Wake Forest School of Medicine, Winston-Salem, NC, United States; 7 Department of Pediatrics, Medical University of South Carolina, Charleston, SC, United States; 8 Biomedical Informatics Center, Medical University of South Carolina, Charleston, SC, United States

**Keywords:** telemedicine, survey, patient preferences, direct-to-consumer telemedicine, patient-provider relationship, inequity, consumer, patient experience, willingness, income, association, satisfaction, mobile phone

## Abstract

**Background:**

A 2017 survey of patient perspectives showed overall willingness and comfort to use telemedicine, but low actual use. Given recent growth and widespread exposure of patients to telemedicine, patient preferences are likely to have changed.

**Objective:**

This study aimed to (1) identify demographic trends in patient preferences and experiences; (2) measure ease of use and satisfaction of telemedicine; and (3) measure changes in telemedicine use, willingness, and comfort since 2017.

**Methods:**

We replicated a 2017 study with a nationwide survey of US adults. The survey, an extended version of the previous study, measured patient health care access as well as knowledge, experiences, and preferences regarding telemedicine encounters. We recruited participants using SurveyMonkey Audience in July 2022. We used descriptive statistics and generalized estimating equations to measure change and identify trends.

**Results:**

We accrued 4577 complete responses. Patient experience with telemedicine was substantially higher in 2022 than in 2017, with 61.1% (vs 5.3%) of participants aware that their primary care provider offered telemedicine and 34.5% (vs 3.5%) reporting use of telemedicine with their primary care provider. This study also reported ease of use and satisfaction rates to be similar to in-person visits, while overall willingness and comfort in using telemedicine increased from 2017. Individuals at the poverty line were significantly less likely to report satisfaction with telemedicine visits. We found increased interpersonal distance in a patient and health care professional relationship significantly reduced patient ease of use, willingness, and comfort in using telemedicine.

**Conclusions:**

This study identified an association between income and patient satisfaction, conveying the importance of understanding telemedicine in relation to health care access and equity. Telemedicine ease of use and satisfaction were comparable to in-person visits. Individuals reported greater use and higher positive perceptions of telemedicine willingness and comfort since 2017.

## Introduction

Telemedicine has allowed for the growth of new technologies and platforms for asynchronous and synchronous health care. Most patients, though, imagine direct-to-consumer, synchronous visits when considering “telemedicine” [1]. While lower costs and higher ease of access had long been recognized in the biomedical literature [2,3], telemedicine did not come into widespread use until the 2020 pandemic caused by SARS-CoV-2. Public health recommendations to use telemedicine greatly expanded its use and created opportunities for expanded research measuring stakeholder satisfaction, as well as barriers and facilitators [4,5]. Given the profound increase in use, it is necessary to understand whether patient preferences, experiences, and needs related to telemedicine have changed. A current understanding of the patient experience can help us to effectively develop and implement telemedicine programs within health care, especially with the push toward hybrid care [6-8].

According to the 2021 National Center for Health Statistics survey, more than one-third (37%) of US adults had used telemedicine [9]. The same study showed demographic trends that may point to differences in preferences or even unequal access to telemedicine based on income, gender, and race [9]. Though studies generally show high satisfaction of telemedicine use among patients [10-12], specific measurement of patient comfort in using telemedicine and willingness to use telemedicine is difficult to find in literature, and most studies were conducted before widespread adoption of telemedicine in the year 2020 [13-15]. Our team, Welch et al [14], conducted a 2017 study measuring aspects of willingness and comfort in direct-to-consumer telemedicine. In a nationwide representative sample of over 4300 US adults, we found that only 5.3% of patients knew their primary care health care professional (PCP) offered telemedicine and 3.5% had ever used telemedicine for their PCP visits [14]. Overall, over 50% of patients were willing and comfortable to use telemedicine with their own PCP [14]. Health care professional scenario types appeared to affect patient willingness and comfort, though: for example, patients were mostly willing (51.9%) to see their own PCP using telemedicine but less so for a different PCP within the health organization (34.9%) or a different PCP from a different organization (18.6%). Similar results were seen regarding comfort with 53.7% of patients being comfortable in using telemedicine with their own PCP but only 18.6% with a different PCP from a different organization [14]. Additionally, over 56% of patients reported that having an established relationship with a health care professional before having a telemedicine visit is important [14]. While this survey study did not include direct measures regarding satisfaction and ease of use, it showed that patients in the United States had a positive disposition toward telemedicine, even with little direct telemedicine exposure. Inclusion of additional assessments within this survey, including measures of satisfaction, would have helped to determine a more holistic understanding of patient preferences and experiences in telemedicine.

Telemedicine satisfaction has been assessed in myriad ways, from brief questions to full-length, validated questionnaires [10,16-19]. Generally, literature shows telemedicine satisfaction can be reliably measured through survey research, comparing satisfaction of telemedicine and in-person visits [17,19]. This approach to understanding telemedicine preferences can reasonably be extended to determine satisfaction with telemedicine in a hybrid care environment. For example, while Welch et al [14] may have measured willingness and comfort in using telemedicine, the addition of specific patient satisfaction questions for both telemedicine and in-person visits would have provided an additional dimension in assessing patient preferences [17]. While previous studies have shown little difference in satisfaction between in-person and telemedicine visits for specific types of visits or specialties [12,20,21], consumer experience with telemedicine has grown enormously in recent years, and their perspectives may have changed [22,23].

One of the largest changes to telemedicine occurred within the realm of compliance and regulation in 2020. With the public health emergency providing the flexibility necessary to implement telemedicine in a widespread fashion [24], telemedicine was provided to the US population in ways previously impossible. Patients could visit with health care professionals across state lines, use modes of telemedicine that were not necessarily Health Insurance Portability and Accountability Act (HIPAA)–compliant, and have the cost of telemedicine visits covered by insurance or other payors [24]. Indeed, with the new flexibilities and regulatory changes, many long-standing barriers to telemedicine usage were placed in the spotlight for public discussion. For example, 2015 guidelines from the American College of Physicians promoted that a patient first meets their health care professional in person prior to any telemedicine visits [25]. Loosened regulations in 2020 placed these expectations under debate [26]. With new flexibilities due to the public health emergency, patients could still access telemedicine if they were unable or unwilling to first meet a health care professional in person [26]. Compliance and regulatory shifts have resulted in critical changes to pathways of telemedicine implementation. As a result, revisiting patient experiences, preferences, and satisfaction with telemedicine is critical in determining ideal implementation strategies of telemedicine. Additionally, understanding patient perceptions of comfort and satisfaction regarding telemedicine may help identify needed changes to national and state policies relating to successful telemedicine practice within the United States.

In prior work, the 2017 Welch et al [14] study, we assessed patients’ willingness and comfort to use telemedicine. The results may be used as a baseline to determine how patient willingness and comfort to use direct-to-consumer telemedicine have changed since 2017, and after large-scale population exposure to telemedicine. Here, we conducted a survey of the US population, replicating the items used in the prior study, to understand how comfort and willingness to use telemedicine has changed. This study aimed to (1) identify potential demographic trends in US patient preferences and experiences in telemedicine visits; (2) measure the current state of patient ease of use and satisfaction with using telemedicine; and (3) measure change regarding telemedicine use, comfort, and willingness among patients since 2017. By assessing the current patient experience in finer resolution, we can better understand patient needs, preferences, and potential barriers to successful use of telemedicine.

## Methods

### Sample and Procedures

We conducted a cross-sectional survey of a national sample of adults from July 1 to July 2, 2022, using SurveyMonkey Audience, an online market research platform [27]. To emulate the Welch et al [14] study, we chose to recruit a similar sample size: we recruited 4500 adults (aged 18 years or older) representative of the US adult population based on age, gender, income, and regionality. Based on sample size calculations, we would be able to report results with a 99.99% confidence level and 2.75% margin of error. The anonymous survey was estimated to take 10 minutes. After providing informed consent, participants answered questions about their PCP, previous use of telemedicine, willingness to use telemedicine, and comfort using telemedicine. Participants received a US $0.50 donation to a nonprofit of their choice in return for their participation. SurveyMonkey Audience uses fraud and bot detection to ensure the integrity of their survey results [27].

### Ethical Considerations

Study procedures were approved as exempt by the Institutional Review Board at the University of South Florida (IRB#4255). Participants were provided with informed consent and were able to opt out of the survey if they chose.

### Survey and Methods

See Multimedia Appendix 1 for a complete list of survey questions and response options. We replicated and extended a previous 2017 survey, developed and reviewed by health care professionals and researchers, to directly compare results [14]. The adapted 10-minute survey comprised 13 multiple-choice questions, which included 7 Likert-scale matrix questions. The outcome measures included participant perceptions of willingness to use telemedicine, comfort in using telemedicine, ease of meeting with health care professionals, and satisfaction in meeting with providers. SurveyMonkey Audience provided 5 multiple-choice screening questions to ensure a representative US sample by gender, age, income, device type, and region.

We retained multiple-choice items from the 2017 survey related to duration (Q1) and frequency (Q2) of visits to a PCP, and added items asking participants of their level of use of telemedicine (Q3) [14]. Next, we asked participants, who responded to having used telemedicine with their PCP, about the satisfaction (Q4), ease of meeting with their health care professionals (Q5), and whether they would be disappointed if telemedicine were no longer offered (Q6). As an extension of the original study, the questions about satisfaction and ease of meeting with providers in person and via telemedicine were anchored on a 5-point Likert scale from 1=very unsatisfied or very difficult to 5=very satisfied or very easy. Additionally, the disappointment question was anchored on a 4-point scale from 1=very disappointed to 5=not disappointed. We then asked participants whether they have used telemedicine with any other health care professionals (Q7) following with the same set of questions regarding satisfaction (Q8), ease (Q9), and disappointment (Q10). All participants were asked about their willingness (Q11) and comfort (Q12) of using telemedicine in different scenario types related to their level of relationship with a PCP (own PCP, different PCP from the same health care organization, and different PCP from different health care organization). Willingness and comfort questions were anchored on 5-point Likert scales similar to the 2017 study from 1=very unwilling or very uncomfortable to 5=very willing or very comfortable. Lastly, we asked participants of their level of agreement (Q13) regarding statements about the importance of (1) having telemedicine as an option, (2) switching to new health care professionals offering telemedicine, (3) having an established relationship with a telemedicine health care professional, and (4) one’s health care professional having access to health records. These importance questions were anchored on a 5-point Likert scale from 1=strongly disagree to 5=strongly agree similar to the 2017 study [14].

### Data Analysis

In order to directly compare the current survey results with the 2017 results, we replicated the analysis detailed in Welch et al [14], using SPSS (version 29; IBM Corp).

We measured each of the four outcomes by the self-reported, 5-point Likert scale; however, for the purposes of analysis, these variables were dichotomized in a similar fashion as seen in Welch et al [14] (eg, very willing, willing, and neutral grouped as willing; and unwilling and very unwilling grouped as unwilling). We reported all four outcome measures in relation to health professional scenario types, income, gender, and age. We determined a reference for each demographic or scenario type for the purposes of analysis (see *Results* section for references).

We computed descriptive statistics for demographic variables and generalized estimating equation (GEE) models for multivariate data modeling of parameter estimates [14,28]. A GEE shows how the average of a response variable of an individual changes with covariates. Additionally, a GEE allows us to view this correlation between repeated measurements on the same individual [29,30].

We used GEE models with the logit function and an exchangeable correlation matrix. For each GEE model, the four demographic predictors were used as independent variables. As the outcome measures were dichotomized, we used binomial logistic models. Independent variables included scenario type (ie, own PCP, different PCP from the same organization, and different PCP from a different organization), age, income, and gender. Each set of independent variables included a reference that yielded adjusted odds ratios (ORs), determining the odds of individuals responding other than the reference category, with the dependent variable in mind (see *Results* section). References were chosen based on the limiting impact of relationship for scenario types and impact of socioeconomic accessibility issues (eg, higher income and age).

## Results

### Overview

We conducted the survey from July 1 to July 2, 2022, and accrued a sample of 4639 participants, resulting in 4577 completed surveys (98.66% completion rate).

### Patient Demographics and Preferences

We present complete descriptive statistics for survey items in Table 1. Patient demographics were representative of the national population by gender and age. Household income displayed a top-heavy trend with 31.07% (1422/4577) reporting they make more than US $75,000.

**Table 1.** Sample characteristics (N=4577).

Demographics		Participants, n (%)
**Age (years)**		
	18‐29	1109 (24.2)
	30‐44	1076 (23.5)
	45‐60	1268 (27.7)
	>60	1124 (24.6)
**Sex**		
	Female	2374 (51.9)
	Male	2203 (48.1)
**Household income (US $)**		
	$0-$9.999	356 (7.8)
	$10,000-$24,999	569 (12.4)
	$25,000-$49,999	1020 (22.3)
	$50,000-$74,999	731 (16)
	$75,000-$99,999	505 (11)
	$100,000-$124,999	346 (7.6)
	$125,000-$149,999	203 (4.4)
	$150,000-$174,999	106 (2.3)
	$175,000-$199,999	80 (1.7)
	$200,000+	182 (4)
	Prefer not to answer	479 (10.5)
**Region**		
	East North Central	555 (12.1)
	East South Central	256 (5.6)
	Middle Atlantic	660 (14.4)
	Mountain	333 (7.3)
	New England	224 (4.9)
	Pacific	861 (18.8)
	South Atlantic	844 (18.4)
	West North Central	288 (6.3)
	West South Central	464 (10.1)
**Device type**		
	iOS phone or tablet	2501 (54.6)
	Android phone or tablet	1076 (23.5)
	Windows desktop or laptop	314 (6.9)
	MacOS desktop or laptop	110 (2.4)
	Other	29 (0.6)

Patients reported a length of relationship with their current PCP of less than 6 months (424/4577, 9.26%), 6 months to a year (527/4577, 11.51%), 1 to 3 years (996/4577, 21.76%), 3 to 5 years (574/4577, 12.54%), or 5 years or more (1618/4577, 35.35%). Some patients (438/4577, 9.57%) reported not having a PCP. Patients noted the frequency of visits to a PCP in the last 12 months: 1 time (1033/4577, 22.57%), 2 times (1197/4577, 26.15%), 3 times (752/4577, 16.43%), 4 times (422/4577, 9.22%), 5 or more times (459/4577, 10.03%), or none (714/4577, 15.6%).

Fewer than half of patients (1909/4577, 41.71%) agreed (1288/4577, 28.14%) or strongly agreed (621/4577, 13.57%) that it is important that their current health care professional offers telemedicine visits. Some (1286/4577, 28.10%) agreed (936/4577, 20.45%) or strongly agreed (350/4577, 7.65%) that they would consider switching to a new health care professional, who offers telemedicine. Most (2864/4577, 62.60%) agreed (1843/4577, 40.27%) or strongly agreed (1021/4577, 22.31%) that it is important to have an established relationship with the health care professional they are having a telemedicine visit with. Most patients (3235/4577, 70.68%) agreed (1706/4577, 37.27%) or strongly agreed (1529/4577, 33.41%) that it is important that their health care professional has access to their health records.

### Measures of Satisfaction and Ease of Using Telemedicine

**Figure 1.**
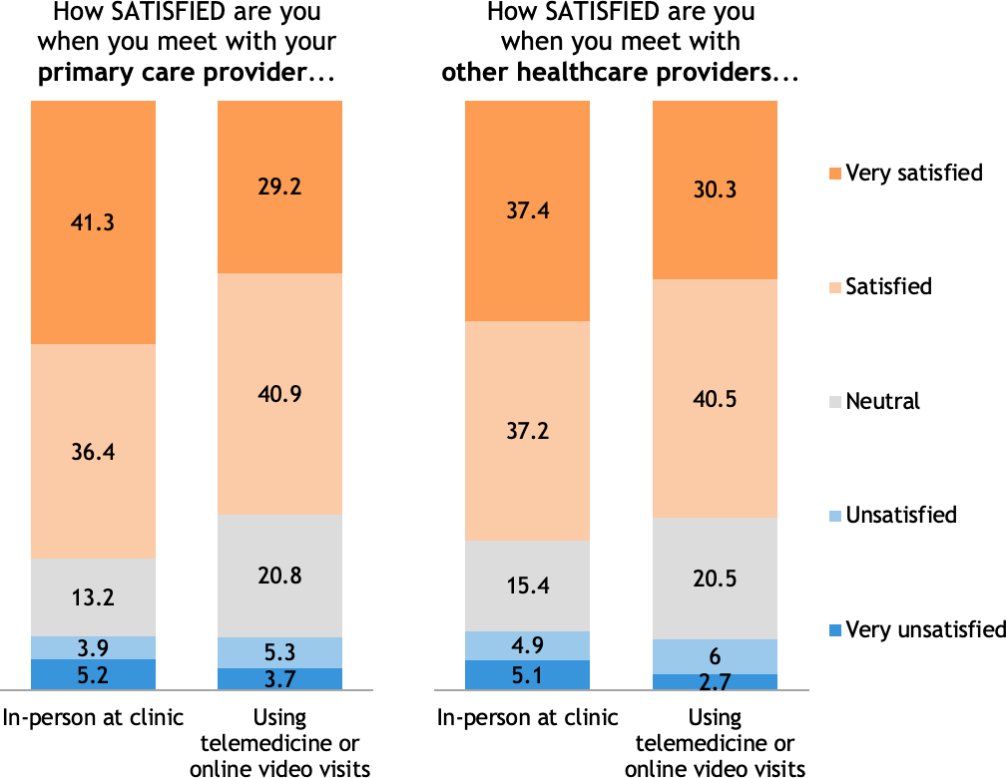
Satisfaction meeting with your PCP and other health care professionals. PCP: primary care health care professional.

**Figure 2.**
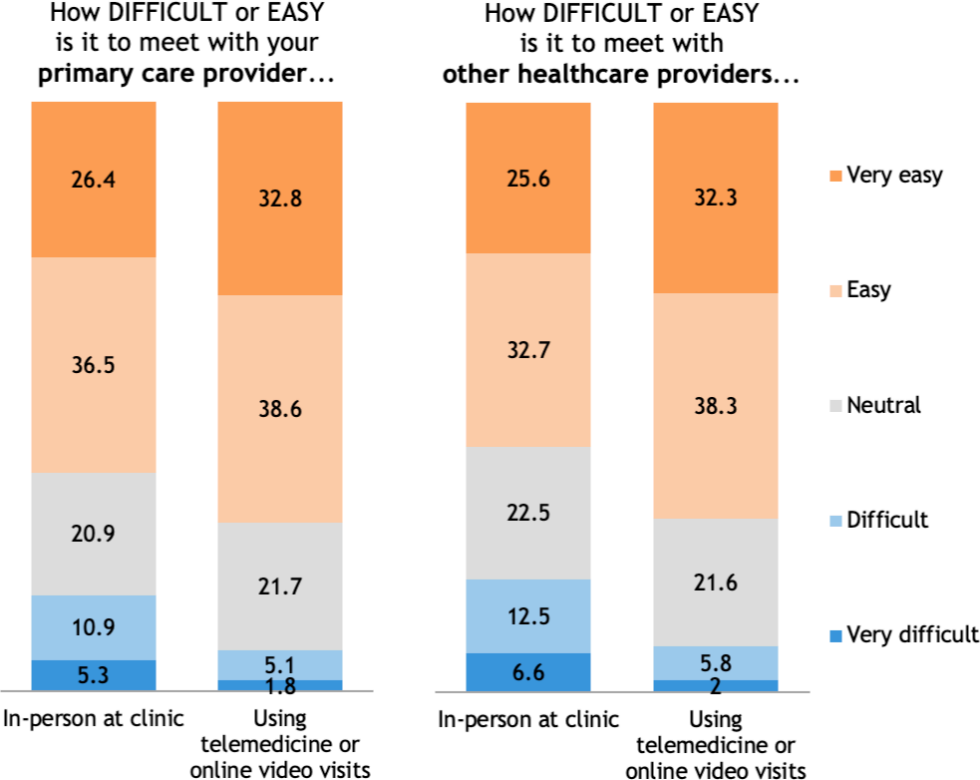
Ease of meeting with your PCP and other health care professionals. PCP: primary care health care professional.

**Figure 3.**
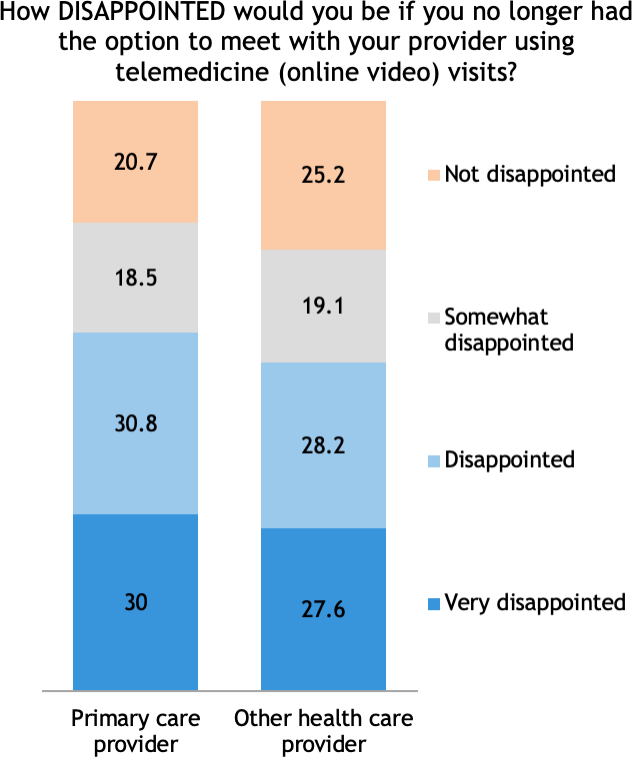
Disappointment if telemedicine was no longer an option to meet with your health care professional.

About 35% (1588/4577) of patients reported having had a telemedicine visit with their PCP and 26.59% (1231/4577) reported that their PCP offers telemedicine, but they have not had a telemedicine visit. Of these, 70.34% (1117/1588) reported being satisfied (651/1588, 40.99%) or very satisfied (466/1588, 29.35%) with telemedicine for online video visits, and 77.83% (1236/1588) reported feeling satisfied (578/1588, 36.40%) or very satisfied (658/1588, 41.44%) with in-person visits at clinic (Figure 1). Regarding ease and difficulty of visits, 71.28% (1132/1588) reported telemedicine visits as being easy (612/1588, 38.54%) or very easy (520/1588, 32.75%), and 62.9% (999/1588) reported seeing their health care professional in person at a clinic as easy (581/1588, 36.59%) or very easy (418/1588, 26.32%; Figure 2). If they no longer had the option to meet with their PCP using telemedicine, 69.9% (1110/1588) reported that they would be somewhat to very disappointed (Figure 3). Some patients reported that their PCP did not offer telemedicine as an option (551/4577, 12.04%), or that they were not sure whether their PCP offered telemedicine (974/4577, 21.28%).

About one-third (1513/4577, 33.06%) of patients reported having had a telemedicine visit with any health care professional (eg, mental health professional, cardiologist, or dermatologist), while 25.54% (1169/4577) reported that their other health care professional offered telemedicine services, but that they had not had a telemedicine visit. Of the 33.06% (1513/4577) that have had a telemedicine visit, 70.79% (1071/1513) reported being satisfied (612/1513, 40.45%) or very satisfied (459/1513, 30.34%) with telemedicine visits, and 74.55% (1128/1513) reported being satisfied (562/1513, 37.14%) or very satisfied (566/1513, 37.41%) with in-person visits at the clinic (Figure 1). Regarding ease and difficulty of visits, 70.65% (1069/1513) reported telemedicine visits with other health care professionals as being easy (581/1513, 38.40%) or very easy (488/1513, 32.25%), and 58.36% (883/1513) reported seeing their other health care professionals in person at a clinic as easy (497/1513, 32.84%) or very easy (386/1513, 25.51%; Figure 2). If they no longer had the option to meet with their other health care professionals using telemedicine, 72.44% (1096/1513) reported that they would be somewhat to very disappointed (Figure 3). Some patients reported that other health care professionals did not offer telemedicine as an option (683/4577, 14.92%) or that they were not sure if their other health care professionals offered telemedicine (1255/4577, 27.42%).

We fitted GEE models for patients who had experienced telemedicine visits and considered scenario type, age, gender, and income. These models suggested patients had lower odds of reporting ease when meeting in person with an established health care professional or new professional from the same organization compared to a telehealth visit with a new provider from a different organization (Table 2). Patients aged between 18‐29 years (OR 0.51, 95% CI 0.37 to 0.71, *P*<.001) and 30‐44 years (OR 0.61, 95% CI 0.44 to 0.84, *P*=.003) had lower odds of reporting ease when meeting in person with their PCP, when compared to patients aged 60 years and older. Individuals who reported an income of US $0‐$24,999 had lower odds of reporting ease when meeting in person with their PCP (OR 0.69, 95% CI 0.50 to 0.95, *P*=.02) as compared to those with an income of US $100,000 or over. Gender did not appear to impact ease of meeting with a health care professional.

Patients reporting poverty-level income (US $0‐$24,999) had lower odds of reporting satisfaction with in-person health care professional meetings when compared to those with an income of US $100,000 or more (OR 0.53, 95% CI 0.37 to 0.75, *P*<.001). Scenario type (eg, own PCP), gender, and age had no statistically significant impact on reported satisfaction in meeting with health care professionals.

**Table 2.** Odds ratios from generalized estimating equation models predicting ease to meet with health care professional and satisfaction of using telemedicine.

		Ease to meet with health care professional		Satisfaction meeting with health care professional	
		Odds ratio (95% CI)	*P* value	Odds ratio (95% CI)	*P* value
**Scenario**					
	PCP^a^—in person	0.404 (0.32 to 0.51)	<.001	0.96 (0.76 to 1.23)	.75
	PCP—telehealth	1.097 (0.85 to 1.41)	.47	0.97 (0.78 to 1.21)	.79
	Other health care professional—in person	0.344 (0.28 to 0.43)	<.001	0.86 (0.69 to 1.07)	.17
	Other health care professional—telehealth	Reference	—^b^	Reference	—
**Income (US $)**					
	$0‐$24,999	0.69 (0.50 to 0.95)	.02	0.53 (0.37 to 0.75)	<.001
	$25,000‐$49,999	0.82 (0.60 to 1.12)	.21	0.77 (0.53 to 1.11)	.16
	$50,000‐$74,999	0.94 (0.66 to 1.34)	.74	0.84 (0.58 to 1.22)	.37
	$75,000‐$99,999	0.78 (0.52 to 1.15)	.21	0.74 (0.48 to 1.16)	.19
	$100,000+	Reference	—	Reference	—
**Gender**					
	Male	1.07 (0.86 to 1.33)	.56	0.87 (0.68 to 1.11)	.26
	Female	Reference	—	Reference	—
**Age (years)**					
	18‐29	0.51 (0.37 to 0.71)	<.001	0.83 (0.58 to 1.19)	.31
	30‐44	0.61 (0.44 to 0.84)	.003	0.98 (0.69 to 1.38)	.91
	45‐60	0.81 (0.58 to 1.12)	.20	1.22 (0.87 to 1.71)	.24
	60+	Reference	—	Reference	—

a PCP: primary care health care professional.

b Not applicable.

### Measures of Telemedicine Use, Willingness, and Comfort

**Figure 4.**
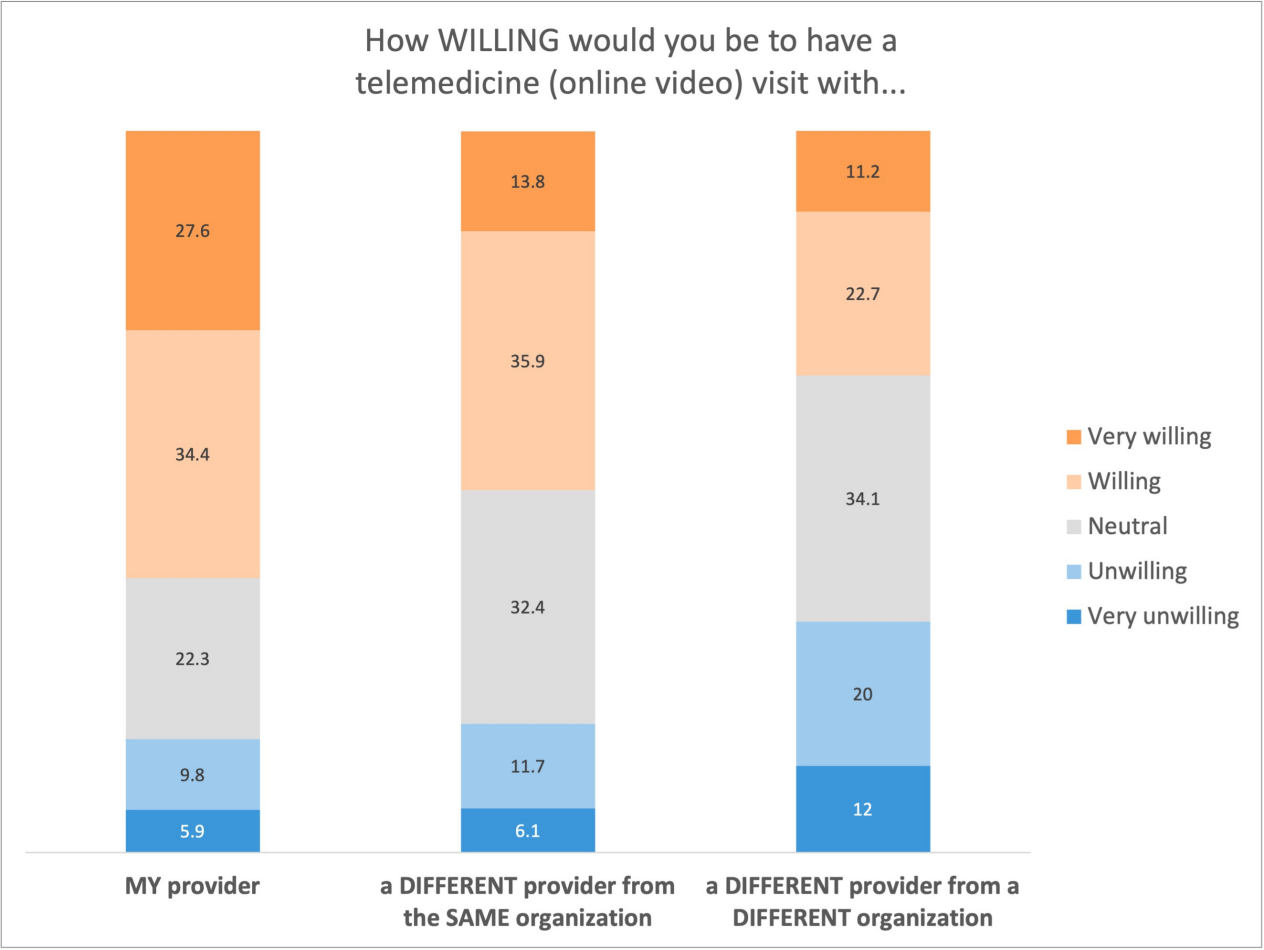
Willingness to have a telemedicine visit.

Over half (2836/4577, 61.96%) of patients would be willing (1573/4577, 34.37%) or very willing (1263/4577, 27.59%) to have a telemedicine visit with their health care professional. About half (2278/4577, 49.77%) would be willing (1645/4577, 35.94%) or very willing (633/4577, 13.83%) to have a telemedicine visit with a different health care professional from the same health care organization. About one-third (1550/4577, 33.86%) would be willing (1037/4577, 22.66%) or very willing (513/4577, 11.21%) to have a telemedicine visit with a different health care professional from a different health care organization (Figure 4).

The odds of patients being willing to use telemedicine with their own PCP (OR 2.49, 95% CI 2.27 to 2.73, *P*<.001) or a different PCP from the same organization (OR 2.21, 95% CI 2.05 to 2.38, *P*<.001) were statistically significantly higher compared to a different PCP from a different organization (Table 3). Age showed an impact on willingness to use telemedicine, with the age group 30‐44 years having the highest odds of willingness compared to those aged 60 and older (OR 2.33, 95% CI 1.96 to 2.76, *P*<.001). Income and gender did not appear to impact willingness to use telemedicine.

**Figure 5.**
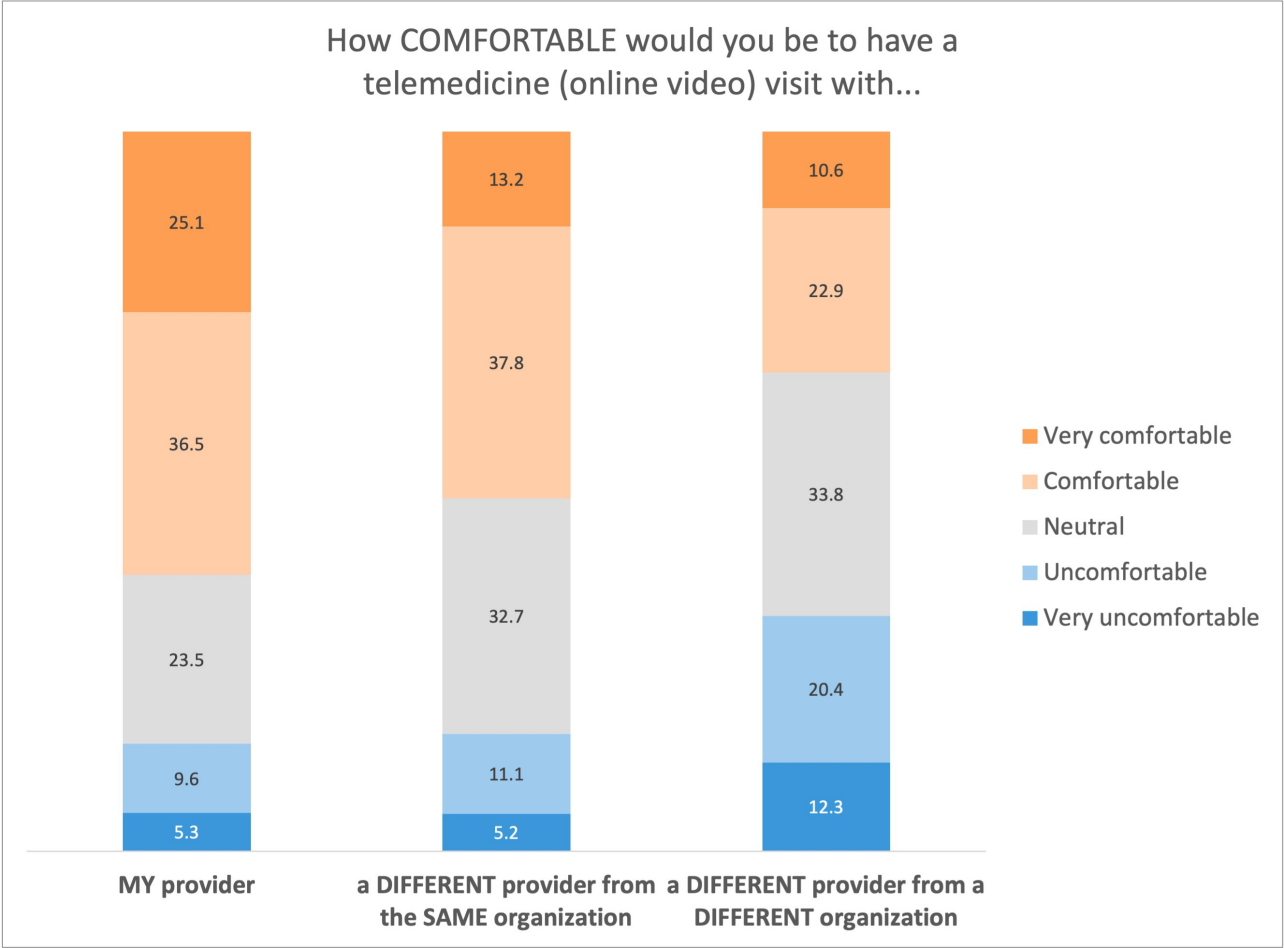
Comfortability having a telemedicine visit.

Most patients (2819/4577, 61.59%) reported being comfortable (1672/4577, 36.53%) or very comfortable (1147/4577, 25.06%) having a telemedicine visit with their health care professional. Most (2333/4577, 50.97%) would be comfortable (1729/4577, 37.78%) or very comfortable (604/4577, 13.20%) having a telemedicine visit with a different PCP in the same health care organization. Approximately one-third of patients (1533/4577, 33.49%) reported being comfortable (1050/4577, 22.94%) or very comfortable (483/4577, 10.55%) having a telemedicine visit with a different health care professional from a different health care organization (Figure 5).

The odds of patients feeling more comfortable in using telemedicine with their own PCP (OR 2.72, 95% CI 2.47 to 2.99, *P*<.001) or a different PCP from the same organization (OR 2.56, 95% CI 2.37 to 2.77, *P*<.001) were significantly higher than those with a different PCP from a different organization (Table 3). Age had a significant effect on comfort in using telemedicine, with the age group of 30‐44 years reporting the highest odds of comfort in using telemedicine compared to those aged 60 years and older (OR 2.35, 95% CI 1.98 to 2.80, *P*<.001). Individuals reporting an income of US $0‐$24,999 had lower odds (OR 0.81, 95% CI 0.67 to 0.97, *P*=.02) of being comfortable using telemedicine in reference to those who made US $100,000 or more. Gender did not appear to affect comfortability in using telemedicine.

**Table 3.** Odds ratios from generalized estimating equation models predicting willingness and comfortability of using telemedicine.

		Willingness to use telemedicine		Comfort using telemedicine	
		Odds ratio (95% CI)	*P* value	Odds ratio (95% CI)	*P* value
**Scenario**					
	Own PCP^a^	2.49 (2.27 to 2.73)	<.001	2.72 (2.47 to 2.99)	<.001
	Different PCP or same organization	2.21 (2.05 to 2.38)	<.001	2.56 (2.37 to 2.77)	<.001
	Different PCP or different organization	Reference	—^b^	Reference	—
**Income (US $)**					
	$0‐$24,999	0.97 (0.81 to 1.17)	.76	0.81 (0.67 to 0.97)	.02
	$25,000‐$49,999	0.95 (0.79 to 1.13)	.53	0.93 (0.77 to 1.11)	.40
	$50,000‐$74,999	1.02 (0.84 to 1.23)	.84	0.96 (0.79 to 1.17)	.70
	$75,000‐$99,999	1.01 (0.82 to 1.25)	.90	0.92 (0.74 to 1.13)	.42
	$100,000+	Reference	—	Reference	—
**Gender**					
	Male	0.99 (0.88 to 1.12)	.91	1.07 (0.94 to 1.21)	.30
	Female	Reference	—	Reference	—
**Age (years)**					
	18‐29	1.61 (1.37 to 1.90)	<.001	1.81 (1.53 to 2.13)	<.001
	30‐44	2.33 (1.96 to 2.76)	<.001	2.35 (1.98 to 2.80)	<.001
	45‐60	1.94 (1.65 to 2.29)	<.001	1.97 (1.67 to 2.32)	<.001
	60+	Reference	—	Reference	—

a PCP: primary care health care professional.

b Not applicable.

## Discussion

### Principal Results

This study aimed to assess demographic trends in patient preferences in telemedicine visits; measure patient ease of use and satisfaction in using telemedicine; and measure changes in patient use, willingness, and comfort with telemedicine since 2017. The results showed that telemedicine has become an accepted part of many people’s health care. We found individuals reporting incomes at or below poverty levels tended to find telemedicine more difficult to use, were less comfortable using it, and were ultimately less satisfied with a telemedicine visit. The level of patient–health care professional relationship (scenario types) significantly affected willingness and comfort in using telemedicine, with closer relationships showing higher levels of comfort and willingness to use telemedicine. This study leveraged the baseline data from a study published in 2017 to understand current patient preferences and the potential impact of increased exposure to telemedicine [14].

### Comparison With Prior Work

We compared public use, knowledge, and perceptions of telemedicine from 2017 and 2022. Importantly, patient telemedicine preferences and knowledge had changed dramatically with 61.1% of patients knowing their PCP offered telemedicine compared to 5.3% in 2017 and 34.69% had used telemedicine with their PCP as compared to only 3.5% in 2017 [14]. Interestingly, nearly a quarter (21.28%) and over a quarter (27.42%) of patients did not know whether their PCP or other health care professionals, respectively, offered telemedicine. While only 12.04% and 14.92% of patients had PCPs or other health care professionals not offering them telemedicine, these numbers contrast greatly when noting that 67.7% of patients in 2017 reported that their PCPs did not offer telemedicine at all. Though an increase was expected, these numbers highlight the drastic increase telemedicine use saw in a short period of time. To put these numbers in perspective regarding general health care visits, most (69.65%) individuals reported being with their PCP for at least one year, and nearly half (48.72%) went to their PCP annually or biannually. While little change was seen in general health care access from 2017, major changes were indeed visible in telemedicine knowledge and use [14].

Overall, satisfaction in the use of telemedicine was found to be comparable (70.34%) to visits in person (77.83%) with a PCP. While in-person visits trended to be higher in satisfaction, telemedicine visits trended to be higher (71.28%) in ease for visits with a PCP than in-person visits (62.90%). About 70% of patients reported they would feel some level of disappointment should their PCP no longer offer telemedicine options. These results conveyed that while people view telemedicine visits similarly to in-person visits regarding satisfaction and ease, having the option for telemedicine access is now expected. Patients and health care professionals now use telemedicine as an additional feature, intervention mode, or aspect of treatment [31].

While the health care professional scenario type impacted the odds of individuals reporting higher ease of using telemedicine for visits, this study indicated patients also generally find telemedicine visits to be easier than in-person visits. Interestingly, a similar result is not seen for the satisfaction measure. Indeed, while age and income had a significant impact on whether patients found telemedicine easier or harder, GEE models showed that patients with an income of US $0‐$24,999 were the only group significantly more likely to report lower satisfaction with health care professional visits when compared to the reference group. Such a result is not unexpected, as those at or below the poverty line would have the most difficulty in accessing telemedicine for health care visits due to the costs associated with having the right hardware (computer, tablet, or smartphone), access to reliable internet, and availability of privacy for a visit [32,33].

Literature supports this study’s results that patients at poverty level incomes are less comfortable using telemedicine, find telemedicine more difficult to use, and are less satisfied with telemedicine than those in higher income brackets. Though telemedicine increases access to health care for many [32], Curtis et al [33] show that digital access disparities can exacerbate current health care inequalities, leaving those most vulnerable even further behind. While telemedicine can be provided from the comfort and convenience of one’s home [34], such a scenario does not explicitly mean a patient is comfortable to use telemedicine [35,36]. For example, a patient who may need to borrow a smartphone for a telemedicine visit may feel uncomfortable asking for the device. While some studies have shown that both health care professionals and patients feel telemedicine may improve access to care due to convenience, there is a need for better assessment of actual patient satisfaction, comfort, and willingness in using telemedicine [14,37]. Considering that the US $0‐$24,999 income group was significantly more likely to report difficulty in meeting with a PCP over telemedicine, this study clearly shows the limitations individuals face due to economic status. Understanding such patient preferences informs approaches to increase telemedicine accessibility [35].

While the development of the patient–health care professional relationship is a critical part of medicine, it is even more scrutinized in telemedicine [38-40]. Though federal laws allow health care professionals to establish a relationship with a new patient via telemedicine, some states pose specific stipulations that severely restrict this process [41]. The three scenarios used in the patient survey provide insight into key aspects of the patient–health care professional relationship—necessary insight for context of how telemedicine is provided in terms of visit environment and communication. The importance of the patient-provider relationship scenarios became evident when looking at reported willingness and comfort in using telemedicine by patients. The results highlighted that people are significantly more willing and comfortable in using telemedicine with their own PCP or at least a PCP from their health organization than an unknown PCP from a different organization altogether. Welch et al [14] found that patients became less willing to use telemedicine as they became further detached from their own health care professional in 2017, our study showed a similar trend for both willingness and comfort. While trust in health care professionals may be measured in many ways, the Trust in Physician scale has shown that patient trust increased with length of relationship with a provider and that patients who were able to choose their providers exhibited higher trust scores [42]. One study by Orrange et al [43] surveyed 368 patients during March-April 2020 regarding satisfaction, trust, and concerns related to telemedicine visits. Patients reported being very satisfied (47.4%) or satisfied (35.3%) with their telemedicine visits, significant correlation with Trust in Physician scores and technical issues, concerns over privacy and cost, satisfaction with convenience, and the amount of time spent in a visit [43]. As the interpersonal patient–health care professional relationship becomes further scrutinized with the new era of telemedicine, knowledge of patient perceptions regarding satisfaction and trust are important for successful implementation of telemedicine and telemedicine policy. The future of health care seems to include a combination of telemedicine and in-person care access, which means improving telemedicine with such factors in mind are key for success.

Though general trends show that willingness and comfort in using telemedicine have increased since 2017, the level of change may provide insight toward actual telemedicine access. Individuals were more likely to report greater comfort and willingness to use telemedicine depending on their age, income, and scenario type. While income did not factor into willingness to use telemedicine, individuals reporting an income at or below poverty levels were significantly less likely to be comfortable using telemedicine. Some literature shows that income may not relate to certain aspects of comfort such as technological literacy; yet, there is a definite need in understanding how income inequalities may affect feelings of comfort [44]. Though all patients may be equally willing to try telemedicine based on income, differences in comfort highlighted current limitations for telemedicine visits in the United States. For example, while patients may want to use telemedicine, they may find themselves uncomfortable in using the technology or asking others for aid [45]. Such differences between willingness and comfort levels have become easier to see: in the 2017 study, willingness and comfort mirrored one another, while this study saw a distinct separation of the two when looking at the comfort of using telemedicine with a wholly new health care professional—18.6% for both comfort and willingness in 2017, and 33.49% comfort and 33.86% willingness now [14]. To implement telemedicine successfully, stakeholder experience and needs must be considered (eg, why is patient comfort lagging behind willingness to use telemedicine?) to make changes in policy and organizational processes.

Prior studies have shown the importance of the patient–health care professional relationship in patient satisfaction and trust [43]. This study showcased that satisfaction and willingness are affected by this relationship and bolstered prior findings. This knowledge is critical in developing successful telemedicine guidelines for health care professionals and patients. As changes to policy and regulation occur for telemedicine [46], a careful consideration of patient preferences and needs would help develop more effective, timely policies. With the knowledge that health care is moving toward a hybrid care model, successful implementation of telemedicine requires that both patient and health care professional preferences are considered. The public health emergency gave telemedicine more regulatory flexibility for both stakeholders, thus helping to lead to the sustained use of telemedicine [47]. Now, policy makers are under pressure to ensure patient and health care professional needs are being met regarding telemedicine and ensuring patient safety and health information security. Understanding that more of the population feel telemedicine is important (41.71% from 19.8% in 2017) showed that telemedicine is now an expected and significant part of health care.

### Limitations

We obtained a national sample using SurveyMonkey Audience, in part to emulate sampling from the 2017 study. However, the platform no longer includes race or education as part of reported demographics or as sampling criteria. Without data describing race and education, it is difficult to compare or fully understand socioeconomic differences between the original 2017 survey and this study. However, we remained able to examine age, gender, and income in relation to willingness, comfort, satisfaction, and ease of use of telemedicine within the United States. It is important to note that though sampling was carried out using the same methods for the purpose of consistency, bias is possible in online survey recruitment platforms. For example, those surveyed already have access to technology in the forms of the devices used and internet. This study leverages the 2017 results as baselines where possible and relevant. As a result, not all methods and analyses reflect the prior study as descriptive and frequency statistics were determined to provide a satisfactory mode for comparison. Future work should include further measures of socioeconomic status, such as race, ethnicity, and education, to further understand how telemedicine is currently used and what can be carried out to improve its accessibility and levels of success.

### Conclusion

With the increased public exposure to telemedicine, there has been a visible difference in telemedicine use and perceptions among the US population. Willingness and comfort to use telemedicine have increased since 2017. Further, the patient–health care professional relationship appears to influence willingness, comfort, and ease of using telemedicine. Additionally, this study highlighted the significant negative effect income has for individuals regarding comfort, satisfaction, and ease of use of telemedicine; this result is especially important when considering that telemedicine is touted as a breakthrough for health care access. Overall, there has been a large increase in telemedicine usage since 2017, with more finding telemedicine an important part of their health care.

## Supplementary material

10.2196/51056 Multimedia Appendix 1 Survey questions.

## Abbreviations

GEE: generalized estimating equation
HIPAA: Health Insurance Portability and Accountability Act
OR: odds ratio
PCP: primary care health care professional
