# Supplementary material for: Patient Preferences for Direct-to-Consumer Telemedicine Services: Replication and Extension of a Nationwide Survey
Source: JMIR Hum Factors. 2024 Nov 27;11:e51056. doi: 10.2196/51056 (PMC11612525; doi:10.2196/51056)
Supplement: Multimedia Appendix 1 [file humanfactors-v11-e51056-s001.docx]

To begin, we'd like to know a little about you and your primary care provider. Your PRIMARY CARE PROVIDER is any clinician (medical doctor, physician assistant, nurse practitioner, etc.) who you typically visit first for an undiagnosed health concern and continuing care of medical conditions.

**1.) How long have you been going to your current primary healthcare provider?**

1. I don't have a primary healthcare provider
2. Less than 6 months
3. 6 months to 1 year
4. 1 to 3 years
5. 3 to 5 years
6. 5 years or more

**2.) In the last 12 months, how many times did you visit your primary healthcare provider?**

1. None
2. 1
3. 2
4. 3
5. 4
6. 5 to 9
7. 10 or more

Telemedicine is a way for your health care provider (such as your primary care provider) to visit with you online using video. With telemedicine, you can meet with your healthcare provider from anywhere.

**3.) Does your current primary care provider offer telemedicine (online video) visits as an option to meet with them?**

1. Yes and I HAVE had a telemedicine (online video) visit with them
2. Yes but I HAVE NOT had a telemedicine (online video) visit with them
3. No
4. I'm not sure
5. I don’t have a primary care provider

*Conditional on a response of 1 to Question 3:*

**4.) How SATISFIED are you when you meet with your provider**

|  | Very unsatisfied | Unsatisfied | Neutral | Satisfied | Very Satisfied |
| --- | --- | --- | --- | --- | --- |
| in-person at clinic |  |  |  |  |  |
| using telemedicine or online video visits |  |  |  |  |  |

**5.) How DIFFICULT or EASY is it to meet with your provider**

|  | Very difficult | Difficult | Neutral | Easy | Very Easy |
| --- | --- | --- | --- | --- | --- |
| in-person at clinic |  |  |  |  |  |
| using telemedicine or online video visits |  |  |  |  |  |

**6.) How DISAPPOINTED would you be if you no longer had the option to meet with your provider using telemedicine (online video) visits?**

- Very disappointed
- Disappointed
- Somewhat disappointed
- Not disappointed

**7.) Do ANY of your other healthcare providers (e.g., mental health provider, cardiologist, dermatologist) offer telemedicine (online video) visits as an option to meet with them?**

1. Yes, and I HAVE had a telemedicine (online video) visit with them
2. Yes, and I HAVE NOT had a telemedicine (online video) visit with them
3. No
4. I'm not sure

*Conditional on a response of 1 to Question 7:*

**8.) How SATISFIED are you when you meet with your provider**

|  | Very unsatisfied | Unsatisfied | Neutral | Satisfied | Very Satisfied |
| --- | --- | --- | --- | --- | --- |
| in-person at clinic |  |  |  |  |  |
| using telemedicine or online video visits |  |  |  |  |  |

**9.) How DIFFICULT or EASY is it to meet with your provider**

|  | Very difficult | Difficult | Neutral | Easy | Very Easy |
| --- | --- | --- | --- | --- | --- |
| in-person at clinic |  |  |  |  |  |
| using telemedicine or online video visits |  |  |  |  |  |

**10.) How DISAPPOINTED would you be if you no longer had the option to meet with your provider using telemedicine (online video) visits?**

- Very disappointed
- Disappointed
- Somewhat disappointed
- Not disappointed

**11.) If you were not feeling well and it wasn’t an emergency, and you had the option to meet with a provider using telemedicine (online video) instead of traveling to a clinic, how WILLING would you be to:**

|  | Very Unwilling | Unwilling | Neutral | Willing | Very Willing |
| --- | --- | --- | --- | --- | --- |
| have a telemedicine (online video) visit with MY provider |  |  |  |  |  |
| have a telemedicine (online video) visit with a DIFFERENT provider from the SAME healthcare organization |  |  |  |  |  |
| have a telemedicine (online video) visit with a DIFFERENT provider from a DIFFERENT healthcare organization |  |  |  |  |  |

**12.) If you were not feeling well and it wasn’t an emergency, and you had the option to meet with a provider using telemedicine (online video) instead of traveling to a clinic, how COMFORTABLE would you be to:**

|  | Very Uncomfortable | Uncomfortable | Neutral | Comfortable | Very Comfortable |
| --- | --- | --- | --- | --- | --- |
| have a telemedicine (online video) visit with MY provider |  |  |  |  |  |
| have a telemedicine (online video) visit with a DIFFERENT provider from the SAME healthcare organization |  |  |  |  |  |
| have a telemedicine (online video) visit with a DIFFERENT provider from a DIFFERENT healthcare organization |  |  |  |  |  |

**13.) Select your level of agreement with the following statements**

|  | Strongly Disagree | Disagree | Neutral | Agree | Strongly Agree |
| --- | --- | --- | --- | --- | --- |
| It is important to me that my CURRENT PROVIDER offers telemedicine (online video) visits as an option |  |  |  |  |  |
| I would consider SWITCHING to a new provider who offers telemedicine (online video) visits as an option |  |  |  |  |  |
| It is important to me to have an ESTABLISHED RELATIONSHIP with the provider I'm having a telemedicine (online video) video visit with |  |  |  |  |  |
| It is important to me that the provider I meet with using telemedicine (online video) can ACCESS my health records |  |  |  |  |  |
